# A 15-user quantum secure direct communication network

**DOI:** 10.1038/s41377-021-00634-2

**Published:** 2021-09-14

**Authors:** Zhantong Qi, Yuanhua Li, Yiwen Huang, Juan Feng, Yuanlin Zheng, Xianfeng Chen

**Affiliations:** 1 State Key Laboratory of Advanced Optical Communication Systems and Networks, School of Physics and Astronomy, Shanghai Jiao Tong University, 200240 Shanghai, China; 2 Department of Physics, Jiangxi Normal University, 330022 Nanchang, China; 3 Shanghai Research Center for Quantum Sciences, 201315 Shanghai, China; 4 Collaborative Innovation Center of Light Manipulation and Applications, Shandong Normal University, 250358 Jinan, China

**Keywords:** Optical physics, Nonlinear optics

## Abstract

Quantum secure direct communication (QSDC) based on entanglement can directly transmit confidential information. However, the inability to simultaneously distinguish the four sets of encoded entangled states limits its practical application. Here, we explore a QSDC network based on time–energy entanglement and sum-frequency generation. In total,15 users are in a fully connected QSDC network, and the fidelity of the entangled state shared by any two users is >97%. The results show that when any two users are performing QSDC over 40 km of optical fiber, the fidelity of the entangled state shared by them is still >95%, and the rate of information transmission can be maintained above 1 Kbp/s. Our result demonstrates the feasibility of a proposed QSDC network and hence lays the foundation for the realization of satellite-based long-distance and global QSDC in the future.

## Abstract (web summary)

A 15-user quantum secure direct communication (QSDC) network is experimentally demonstrated, and any two users can perform QSDC task over 40 kilometers of optical fiber.

## Graphical abstract

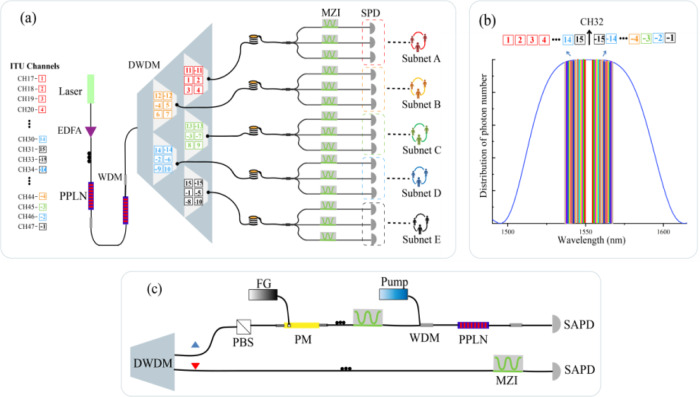

## Introduction

Quantum communication^1^ has presented a revolutionary step in secure communication due to its high security of the quantum information, and many models including quantum key distribution (QKD)^2^, quantum teleportation^3^, and quantum secure direct communication (QSDC)^4^ have been developed. Based on QKD technology, many different types of quantum communication networks have been proposed, such as an eight-user quantum communication network using multiple entangled states in the case of trusted node-free^5^, an entanglement-based wavelength-multiplexed quantum communication network^6^, and an integrated space-to-ground quantum communication network^7^. However, these communication networks based on QKD technology only transmit the key, but do not directly transmit information.

QSDC^8–11^ sends secret information directly over a secure quantum channel. It does not require key distribution and key storage. Any attack of QSDC results in only a random number, and cannot obtain any useful information from it. Therefore, QSDC has simple communication steps and reduces potential security loopholes, and offers higher security guarantees, which continues to enhance the security and the value propositions of quantum communications in general^10^. Recently, the experimental QSDC has been developed significantly. QSDC protocols based on entanglement^8–10^ have been experimentally realized in 0.5 km fiber transmission system^12^, and QSDC has also been demonstrated in the practical experiment by using quantum memory^13^. QSDC based on single photons have been experimentally demonstrated^14^, and a practical working prototype has been implemented^15^. Security analysis of QSDC has been implemented in ref. ^16^. The feasibility of QSDC from geosynchronous Earth orbit satellite to the ground has been demonstrated recently^17^. However, the inability to simultaneously distinguish the four sets of encoded orthogonal entangled states in entanglement-based QSDC protocols limits its practical application. Furthermore, it is important to construct a quantum network in order to make wide applications of quantum secure direct communication. Experimental demonstration of QSDC is badly required.

Here, we present a fully connected entanglement-based QSDC network including five subnets, with 15 users. Using the frequency correlations of the 15 photon pairs via time-division multiplexing (TDM) and dense wavelength division multiplexing (DWDM), we perform a 40-km fiber QSDC experiment by implying two-step transmission between each user without generating secure keys. In this process, we divide the spectrum of the single-photon source into 30 International Telecommunication Union (ITU) channels. With these channels, there will be a coincidence event between each user by performing a Bell-state measurement (BSM) based on the sum-frequency generation (SFG). This allows the four sets of encoded entangled states to be identified simultaneously without post selection, and the fidelity of the entangled photon pair after the SFG is >95%. In our QSDC network, each user can request to communicate with others at any time. The connection relies on distributing entangled photon states between several users, expanding a wider range of applications for further quantum information processing processes.

### Network composition and experimental set-up

We briefly describe the basic process of QSDC which is based on the QSDC protocols of refs. ^8,10^, and the quantum entanglement states used in our work is the time–energy entanglement and SFG. We assume that any two users in the QSDC network want to communicate directly, i.e., User 1 wants to send information to User 2. They share *N* pairs of time–energy-entangled states $\left| {\phi ^ + } \right\rangle = \left( {\left| {ss} \right\rangle + \left| {ll} \right\rangle } \right)/\sqrt 2$, where *s* and *l* indicate that the entangled photons can either travel through a short or a long path. The detailed steps are as follows. (i) Detect the quantum channel to ensure its absolute safety. (ii) They agree that |*ϕ*^+^〉, |*ϕ*^–^〉, |*ψ*^+^〉, and |*ψ*^–^〉 encode the bit values 00, 01, 10, and 11, respectively. $\left| {\phi ^ \pm } \right\rangle = \left( {\left| {ss} \right\rangle \pm \left| {ll} \right\rangle } \right)/\sqrt 2$ and $\left| {\psi ^ \pm } \right\rangle = \left( {\left| {ls} \right\rangle \pm \left| {sl} \right\rangle } \right)/\sqrt 2$ are the four sets of Bell states. (iii) User 1 performs one of four unitary operations *I*, *σ*_*z*_, *σ*_*x*_, or –*iσ*_*y*_ on the photons in his hand, to convert |*ϕ*^+^〉 to |*ϕ*^+^〉, |*ϕ*^–^〉, |*ψ*^+^〉, or |*ψ*^*−*^〉, respectively. These operations correspond to the encoding information 00, 01, 10, and 11, respectively. (iv) User 2 performs the BSM based on SFG to decode the information. This process allows the four sets of encoded Bell states to be identified simultaneously.

**Fig. 1:**
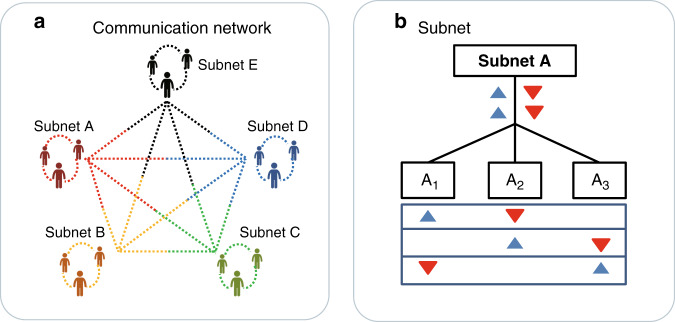
Composition of a quantum network. **a** The quantum network is fully connected by five subnets (A, B, C, D, and E are represented by red, orange, green, blue, and black, respectively). The dotted lines between the subnets (ten links with different colors) are the correlated time–energy photon pairs between the subnets. **b** Every subnet (such as subnet A) is equipped with a 1 × 3 BS and a delay controlling module, which splits a frequency-correlated entangled photon pair (red and blue signs) and sends them to three users randomly.

The complete network composition is divided into two layers as shown in Fig. 1. The left figure illustrates that the network processor provides ten wavelength channels for the combination between subnets so that ten links among the five subnets (A, B, C, D, and E) constitute a fully connected quantum network. Ten time–energy-entangled photon pairs between the subnets are divided into 20 ITU channels (1/−1 to 10/−10) via a 100 GHz DWDM. DWDM is placed in the quantum-network processor, and the output modules of multichannel are connected to users in each subnet. The quantum processor needs to distribute five pairs of entangled photons to realize the interconnection of three users in each subnet (11/−11 to 15/−15), as shown in Fig. 1b. The resulting wavelength channels (that is, subnet A with ITU channels CH17-CH20) are sent to subnet A, which are used to communicate with subnets E, D, C, and B, respectively. Here, the processor allocates a pair of entangled photon pairs to realize the interconnection between the two subnets, such as subnet A and B via 4/−4. At the same time, two subnets respectively share two pairs of entangled photons to connect the three users in their respective subnets, such as A and B via 11/−11 and 12/−12. The sharing of entangled photon pairs and interconnection between subnets are supported by controlling the distribution of four wavelengths from the entanglement source so that each of them will have coincidence events with others. In each subnet, as shown in Fig. 1b, a pair of entangled photons are separated by a passive beam splitter (BS) through TDM, and then randomly distributed to three users. For example, in the structure of subnet A, the upper triangle is the entangled photons of channel CH27, and the opposite triangle is the wavelength channel of CH37. In order to realize the connection among the three users, a time-delay module will be controlled to achieve mutual coincidence events in the subnet. The time-delay module in the subnet is composed of the delay module of detectors and wires, which can distinguish different users in the time domain during the measurement. This way, the different wavelength allocation for 15 users can be realized by wavelength and TDM methods, in order to ensure the complete connection in the network.

**Fig. 2:**
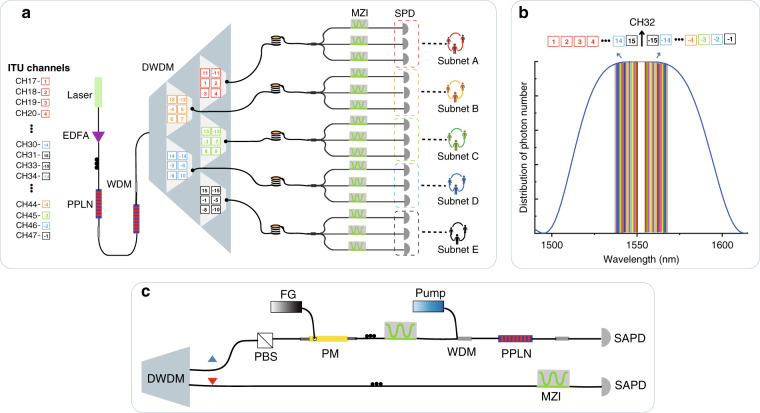
Experimental set-up. **a** The physical structure of the quantum network. The spectrum is split into 30 ITU grid channels via a 100 GHz DWDM. CH17 to CH31 are numbered from 1 to 15, respectively, and the numbers with opposite sign denote corresponding to channels CH33-CH47. The architecture of wavelength allocation is omitted in the small trapezoid multiplex blocks. Each small block with colored digital symbols constructs a wavelength group distributed by the network processor. **b** The blue solid curve is the spectrum of the entangled photon source generated by the SPDC process calculated by the Sellmeir equation^21^. Each pair of signal photon and idler photon is indicated by the same colored bars with and without the opposite digital sign. The *y* axis represents the number of photons corresponding to each channel. **c** Illustration of SFG progress. Photons generated in pairs by SPDC process are multiplexed into the SFG experiment to realize encoding and quantum communication.

To generate SFG photons, we developed an entangled photon source with spontaneous parametric down-conversion (SPDC) process^18–20^ (“Materials and methods”; Fig. 2a). In the experiment, we measured the fidelity of the generated entangled states by correlation records, larger than 97.5% ± 1.0%. It is significant to confirm that the single-photon source can provide high-quality photon pairs with all available ITU wavelength channels, as shown in Fig. 2b, the spectrum of the single-photon source is divided into 30 ITU channels.

Next, we will introduce the SFG encoding process between two users with silicon avalanche photodiodes (SAPDs). In the experiment, we implement a frequency-correlated photon pair after the SPDC process (CH34 and CH30) to realize the SFG process in the network^20,22^ (“Materials and methods”; Fig. 2c). The QSDC process in network is performed in two steps with selected channels CH34 and CH30 (refer to Alice and Bob, respectively). We first establish a secure quantum entanglement channel over 40 km of optical fiber. To guarantee the network security completely, single photons carrying polarization information are transmitted within the form of quantum data blocks. It is generally necessary to use quantum memory in the time domain to effectively control information transmission during QSDC tasks^13^, and the specific function is to adjust the time for storing photons. In our network, when the detection sequence is transmitted, we can achieve the function of quantum storage by adjusting the time delay of the electrical signal through a circuit delay module when the idler photons arrive at the detector. The delay can be adjusted according to the length of the detection sequence. After the safety detection is completed, the communication process is carried out. First, user Alice selects a small part of photons distinguished in time as the detection sequence and sends them to Bob in the quantum channel. Second, Bob has the option to randomly choose the *Z*-basis or *X*-basis to measure the single-photon received by Alice and then, publishes the position of measured photons, the measurement basis, and measurement results through the classical synchronization channel. At the same time, additional wavelength channels can be alternatively blocked that are not needed for communication. Third, Alice chooses the same measurement basis as Bob to measure the corresponding entangled photons, and then she compares the measurement results with the information informed by Bob and estimated the quantum bit error rates (QBERs), which can obtain the secrecy capacity of the system. Once the quantum bit error rate is lower than the threshold, after the comparison of the photon numbers between two users, the channel can be considered to be safe and unattended. In the second stage, the quantum-network processer prepares *N* Bell states with the form of |*ϕ*^+^〉. The form of the state contains the signal photons and idler photons from the entanglement source, and next the entangled photon sequences are separately sent to a pair of users (Alice with *S*_*A*_ and Bob with *S*_*B*_). Once Bob confirms the receipt of the photon sequence (*S*_*B*_), Alice applies different voltages to the polarization modulator (PM)^22^ via a function generator (FG) and directly assigns the information into the *S*_*A*_ sequence. Finally, Bob receives the sequence and then identifies the coded information from Alice through with or without SFG photons. In a realistic QSDC network, users can perform eavesdropping checking in the quantum channel at any time. If the monitored QBER is always lower than the threshold, it is considered that the communication is successful. In this case, each pair of legitimate users can perform block transmission in the second step, and repeat the process until the information transmission is completed.

## Results

**Fig. 3:**
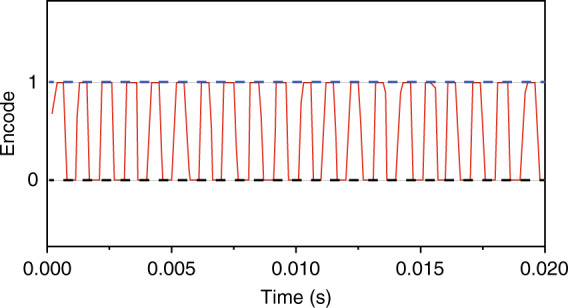
Channel-security detection using single photons. The collected waveform (red) corresponding to the change with the generated SFG photon number is accumulated every 0.02 s while including a bin width of 300 ps. Codes “0” and “1” are indicated by the dotted blue and black lines, respectively.

We performed the channel-security detection in QSDC based on single photons^14,23^ (“Materials and methods”). The first step, as mentioned above, is to ensure the security for the transmission in the quantum channel, and then any two users in the network continue the connection step if the communication environment is demonstrated to be safe^24–27^. Using the FG to apply a square wave voltage with the amplitude of 7 V, which operated at a frequency of 1 kHz, Alice modulates the polarization of the signal single photon (CH33) with a PM. Bob receives the single photon after fiber-based transmission and combines with a 1950-nm pump laser in order to perform upconversion in the periodically poled lithium niobate (PPLN) waveguide. The SAPD is digitized and deliver to the computer, where the photon counts value for coincidence events is used to reflect the result of encoding displayed on a waveform graph, as illustrated in Fig. 3. The maximum number of the generated SFG photons reaches 10^5^ per second. Consequently, the result obtained with the modulation of a single photon rotated from 0° to 90° will directly affect the generation number of SFG photons, which is reflected in the presence or absence of SFG photons. Assume that the assignment of “0” or “1”, it is agreed with the higher and lower level of the waveform graph for clarity, respectively. The collected waveform achieves a perfect performance in the absence of noise with the feedback of modulated information without mistakes. For observing the modulation result clearly, the speed of the PM modulation is adjusted to 1 kHz. However, during the practical detection process, users have an option to adjust the modulation rate via an FG, thereby matching the average number of generated SFG photons. In our experiment, the eavesdropper can only intercept part of the entangled particle in the system, and cannot obtain the overall state of the entangled quantum pair at the same time. That is to say, the action of Eve’s eavesdropping only points to a random polarization state of the photon and limits the resolution of encoded operations, thus ensuring security in the network. Furthermore, the eavesdropping effect will be noticed by users through a significant decrease of the photon number.

**Fig. 4:**
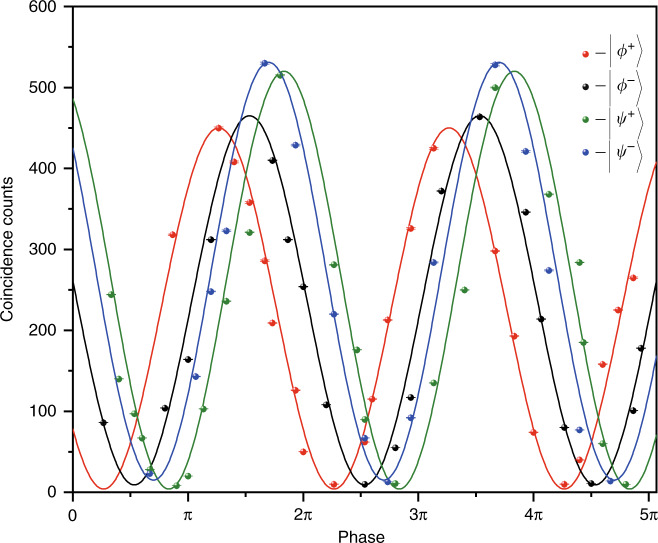
Experiment results. We measured two-photon interference fringe for four Bell states with subtraction of accidental coincidences. Note that the *y* axis represents the coincidence count varied with the full control of the phase over 2π rad.

After the detection, both users share the information when their detectors both clicked by detecting one photon within the coincidence window. In addition, we also conducted the fidelity measurement with four sets of Bell states, as shown in Fig. 4, and the measurement results show that the fidelity of four Bell states is almost as perfect as that before SFG, as listed in Table 1. Here, we can simultaneously distinguish the four Bell states by obtaining the maximum value of the number of generated SFG photons in the two-photon interference fringe. Still, the single-photon characteristics after the SFG do not change.

**Table.1:** Indication of the average fidelity with four Bell states.

Bell-state	Encode	Fidelity
|*ϕ*^+^〉	00	95.25% ± 0.29%
|*ϕ*^–^〉	01	95.43% ± 0.14%
|*ψ*^+^〉	10	95.49% ± 0.13%
|*ψ*^–^〉	11	95.48% ± 0.24%

## Discussion

The results obtained here show the characteristics of a quantum network enabling the use of the SFG process for communication. In what follows, we discuss some of the aspects that are achieved for our experiment, as listed in the following four points. First, four Bell states can be resolved without post selection when the SFG photons are detected, which is different from the linear optical BSM since it can only distinguish two Bell states. Second, by multiplexing the entangled states and connecting to the single-photon detector for individual users, our design can directly provide convenience for several users to interconnect with any others. Users can optionally detect the photons that need for communication and block other wavelength signals without the involvement of the network provider. Moreover, we note that our scheme is also capable of adding or removing users without adding other technologies or altering the entanglement source. As the number of users increases, we can choose to increase ITU international wavelength channels or utilize narrower-band DWDMs. Third, according to the generation rate of SFG photons, we can achieve a faster transmission rate during network communication. It is important to comment that the improvement of the count rate for coincidence events is significantly related to high-performance detector efficiency and a shorter coincidence window. Apart from sources of noise in the measurement, nonideal aspects of PM such as modulation rate, the existence of unmodulated single photon, and the response speed of the detector may contribute to limit the transmission rates and increase the accidental coincidence rates. Taking into account the total loss of the network architecture, we temporarily set 1 kHz rate for single-photon modulation in order to observe the modulate results. The main advantage is that the information transmission rate can be obtained based on the number of generated SFG photons. To generate better enhancement of the transmission rate, we can choose to accelerate the modulation speed to a level that matches the SFG photon generation rate (up to 100 Kbp/s). The alternative methods to increase the number of SFG photons are specific to improve the SFG efficiency (the pump power for frequency conversion) and the quality of detectors (efficiency, response rate, and dark-count rate). Fourth, applying QSDC network to measurement-device-independent QSDC^28,29^ and device-independent QSDC^30^ can relax the security of the internal work of quantum devices, which can not only enhance the security of quantum communication but also effectively improve the quality of communication. It is worth noting the present work, which offers long-distance point-to-point QSDC connection, combined with the recently proposed secure-repeater quantum network of QSDC^31^, which offers secure end-to-end communication throughout the quantum Internet, will enable the construction of secure quantum network using present-day technology, realizing the great potential of QSDC in 6 G future communication^32^.

## Conclusion

We have successfully realized a deterministic QSDC network based on entanglement and SFG with improved scalability. With this scheme, each user interconnects with any others through shared pairs of entangled photons in different wavelengths. Moreover, it is possible to improve the information transmission rate greater than 100 Kbp/s in the case of the high-performance detectors, as well as high-speed control in PM being used. It is noteworthy that this approach can also be implemented for the multiple interconnection networks through setting quantum repeaters^33^. In addition, single-photon frequency conversion via cascaded nonlinear processes^34^ can also be applied to achieve QSDC in large-scale distant networks. The concept of the quantum-network design provides a framework for any realistic quantum communication system, such as quantum teleportation.

## Materials and methods

### Entangled photon source

A continuous-wave pump laser centered at 1551.69 nm (CH32) is used to pump the first temperature-stabilized (24.5 °C) periodically poled lithium niobate (PPLN) waveguide for second harmonic generation^20^ (SHG). The pump laser is connected with an erbium-doped fiber amplifier (EDFA). The noise created by pump power is blocked by using a 780-nm/1550-nm wavelength division multiplexing (WDM). The converted near-infrared laser (775.8 nm) is coupled into the second type-0 PPLN (*T* = 68.5 °C) waveguide with a poling period of 19 μm for SPDC to create the spectrum of frequency-correlated photon pairs located symmetrically about CH32 because of the energy conservation. Each pair of signal photons and idler photons is sent to two unbalanced Mach–Zehnder interferometers (MZIs), and the MZIs are used to analyze and detect entangled photon pairs. Users in the network are allocated a MZI and the detection module, namely a single-photon detector (SPD), while a single-photon detector is an important part of each user. This means that two users will have coincidence events with an SPD attached in the respective detection analysis module. Each detection module used an SPD with a detection efficiency of about 10%, 100 MHz repetition frequency, 1 × 10^–6^ dark-count rate per gate, and 1 ns gate width. To characterize the quality of entanglement, we tested the coincidence-to-accidental ratio (CAR) of entangled photon pairs with the variation of the pump power, as depicted in supplementary with three sets of wavelength channels. In the case of the pump power ≈47 μW, we can obtain CAR ≈500, and hence, the single photons in each channel exhibit basically the same characteristics, forming a stable and controllable single-photon source for generating SFG photons.

### SFG encoding process

In the network, four Bell states with corresponding encoding information are generated by polarization modulator (PM). Then the encoded photons are converted into SFG photons through a nonlinear process to realize the Bell-state measurement. During the process of BSM, distinguishing the four Bell states can be achieved by detecting continuously generated SFG photons. Therefore, the BSM process is completed once the coincidence event between the encoded photon and the corresponding entangled photon is measured.

In our experiment, one of the signal photons (CH34, 1550.12 nm) is connected with the polarizing beam splitter (PBS), and then the PM controlled by the square wave voltage output from the function generator (FG) modulates the polarization of the single photon from horizontal (H) to vertical (V). Then the selected channel and a pump laser with a wavelength of 1950 nm^18^ are coupled into the third PPLN waveguide through a WDM (1550 nm/1900 nm) for SFG. The third PPLN waveguide is poled with a quasi-phase-matching period of 19.6 μm^19^. Based on temperature control to maintain the temperature at 52 °C to achieve phase-matching conditions, the waveguide operated at an efficiency of 55.6% with SFG. The signal photon (CH34) modulated by PM and the idler photon (CH30) pass through two unbalanced MZIs separately, where the MZI is responsible for directing two input photons along either the long or short optical path. We can change the measured basic vector by adjusting the phase of either MZI channel, thus observing the two-photon interference fringes under two noncollinear basic vectors. In terms of receivers, the measurement module consists of a SAPD at the dark-count level and a photon correlator used to record the coincidence counts between two users. The SAPDs have detection efficiencies up to 45% at wavelength 863.6 nm and a dark-count rate ≈25 cps.

### Security analysis

It is well-known that the security and reliability of the information transmission for QSDC is an essential part in the quantum network^12^. Due to the imperfection efficiency of detectors and the loss of the transmission channel, insecure factors are likely to give the eavesdropper opportunities to intercept code information, namely the loss of the signal also means the leakage of information. Therefore, we implemented block transmission and step-by-step transmission methods in QSDC with estimating the secrecy capacity of the quantum channel. After confirming the security of the quantum channel, the legitimate user performs encoding or decoding operations within these schemes reliably, if not, they terminate the communication. In the following, we implemented the wiretap channel model^24^ to calculate the secrecy capacity *C*_*s*_ in the quantum channel, by estimating the QBER threshold, once it is within a certain threshold, the information transmission between legitimate users will continue. Considering the measurement results of quantum states, we can obtain the QBERs (*e*_*x*_ and *e*_*z*_) under two measurement basis vectors, *σ*_*x*_ and *σ*_*z*_, in the eavesdropping checking process. Given the transmission capacity of the main channel between two users, we can get the lower bound of the secrecy capacity^25^

1 $$C_s \ge Q^B\left[ {1 - H\left( e \right)} \right] - Q^EH\left( {e_x + e_z} \right)$$

where *Q*^*B*^ and *Q*^*E*^, respectively, represent the response rate of Bob in the main channel and the maximum probability that Eve can access qubits in the wiretap channel, and *H*(*x*) is the binary Shannon entropy. *e* is the bit error rate of the main channel between Alice and Bob, which can be estimated during the decoding process. Then one can estimate the secrecy capacity *C*_*s*_ using the above Eq. (1). Together with our encoding method, our system yields a bit error rate of 0.0013. According to the calculation in Supplementary Fig. S2, the system can achieve almost perfect secrecy capacity transmission, which also illustrates that the security of information transmission is assured. Moreover, it is also possible to apply the three-party QSDC protocol with hyperentanglement^26^ or masking protocol^27^ to increase the security transmission capacity of QSDC in the network. More details for the security analysis of our quantum network can be found in the Supplementary Material.

## Supplementary information

Supplementary Information for “A 15-user quantum secure direct communication network”

## Data Availability

The supporting data for the findings in this study are available from the corresponding author upon reasonable request.
