# Supplementary material for: A 15-user quantum secure direct communication network
Source: Light Sci Appl. 2021 Sep 14;10:183. doi: 10.1038/s41377-021-00634-2 (PMC8440625; doi:10.1038/s41377-021-00634-2)
Supplement: Supplementary file 1 — Supplementary Information for “A 15-user quantum secure direct communication network” [file 41377_2021_634_MOESM1_ESM.doc]

Supplementary Information for

“A 15-user quantum secure direct communication network”

Zhantong Qi^1, ‡^, Yuanhua Li^1,2,‡^ ,* Yiwen Huang^1,‡^ , Juan Feng^1^ , Yuanlin Zheng^1,3^ , and

Xianfeng Chen^1,3,4^, *

*^1^ State Key Laboratory of Advanced Optical Communication Systems and Networks,*

*School of Physics and Astronomy, Shanghai Jiao Tong University, Shanghai 200240, China*

*^2^ Department of Physics, Jiangxi Normal University, Nanchang 330022, China*

*^3^ Shanghai Research Center for Quantum Sciences, Shanghai 201315, China*

*^4^ Collaborative Innovation Center of Light Manipulation and Applications, Shandong Normal University, Jinan 250358, China*

*‡ These authors contributed equally to this work*

****Correspondence to:***[*xfchen@sjtu.edu.cn*](mailto:xfchen@sjtu.edu.cn)*,* [*lyhua1984@jxnu.edu.cn*](mailto:lyhua1984@jxnu.edu.cn)

**Content**

1. **Coincidence counts between users in the time domain**
2. **Security analysis**
3. **Coincidence counts between users in the time domain**

We measured the coincidence counts of entangled photon pairs in the network, and illustrated the selected subnet with TDM as shown in Fig. S1. The figure also points out that different users can be distinguished by time sequence, including users in the subnet and users between subnets.


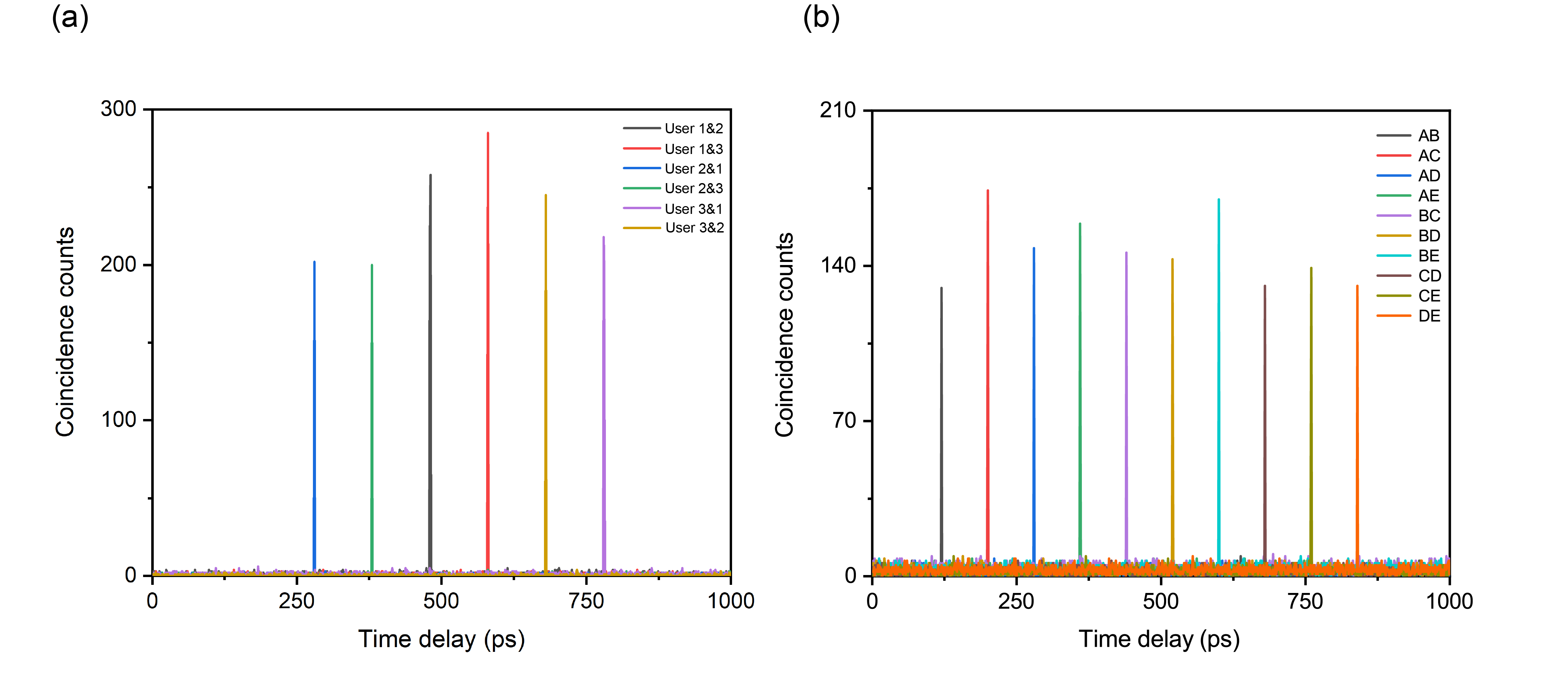


Fig. S1 | Coincidence counts between users in the time domain. (a) Users in the subnet. (b) Users between the subnets.

1. **Security analysis**

Security analysis of information transmission in the quantum channel is important for practical quantum secure direct communication (QSDC) network. The degree of eavesdropping can be estimated before the real QSDC through the judgment of the QBERs threshold^1^. At the same time，we can obtain the quantum bit error rates (QBERs) ($e_{x}$and $e_{z}$) under the two measurement basis vectors, $\sigma_{x}$ and $\sigma_{z}$, in the eavesdropping checking process. If QBERs are within a certain threshold, the user continues to the second step of encoding process. After confirming the security in the quantum channel, the user Alice randomly selects one of the four unitary operations ($I,\text{ }\sigma_{x},\text{ -i}\sigma_{y\text{ }},\text{ }\sigma_{z}$) to perform the encoding scheme, and then sends them to Bob. Bob receives the signal photons and then decodes them by measuring the qubits to obtain the information.

According to Wyner's wiretap channel theory^2^, our QSDC network can be treated in the wiretap channel model. The information transmission of quantum channel is mainly composed of the main channel and the wiretap channel. Here, the main channel represents the quantum channel between the sender and receiver, and the wiretap channel represents the channel between the eavesdropper (Eve) and legitimate users in the network, which also includes channel noise and loss. $C_{s}$ is the maximum secrecy capacity of the Bob-to-Alice (B-A) quantum channel in the first step. Encoded secure communication can be guaranteed if the secrecy capacity $C_{s}$ is positive, it also means that the information capacity $C_{m}$of the main channel is larger than the capacity $C_{e}$ in the wiretap channel. The secrecy capacity is

$C_{s}=C_{m}-C_{e}\text{ }=\max\{I(A:B)-I(A:E)\}$ S(1)

where $I(A:B)$ and $I(A:E)$ represent the mutual information of Alice-to-Bob (A-B) and Alice-to-Eve (A-E) channel respectively.

Eve may intercept qubits and perform measurement operations in the B-A channel, and then resend the qubits to Alice until the qubits are sent back so that Eve can obtain maximal information. Therefore, Eve can perform any measurement operation in A-E channel () jointly. We can get

S(2)

S(3)

where is the Bell state between Alice and Bob, and $|\kappa_{i}\rangle$ is a set of orthogonal states of the eavesdropper 's system. Here we assume $a_{i}\in(00,01,10,11)$.

Similar to the estimation of QKD security code rate^3^, we need to consider the worst case to ensure the security of QSDC. Therefore, it is particularly important to estimate the maximum value of $I(A:E)$. According to Holevo's theorem^4^, we can estimate the upper bound of the classical information under a certain quantum encode approach by using von Neumann entropy

S(4)

where is von Neumann entropy and $\chi$ is the Holevo bound. Based on the formula S(1), the capacity of the main channel can be obtained by estimating the QBER of the information transmission between two users. Thus, we can get the lower bound of the secrecy capacity

$C_{s}\geq Q^{B}[1-H(e)]-Q^{E}H(e_{x}+e_{z})$ S(5)

where $H(x)=-x\log_{2}x-(1-x)\log_{2}(1-x)$ is the binary Shannon entropy. Together with our encode method in the quantum network, our system yields a bit error rate of 0.0013. According to the calculation in the Fig. S2, our system can achieve almost perfect secrecy capacity transmission, which also illustrates that the security of information transmission is assured. Taking into account the imperfect efficiency of detectors and the information transmission with a finite length^5^, the threshold of bit error rate will move forward, which will not affect the security of information transmission.


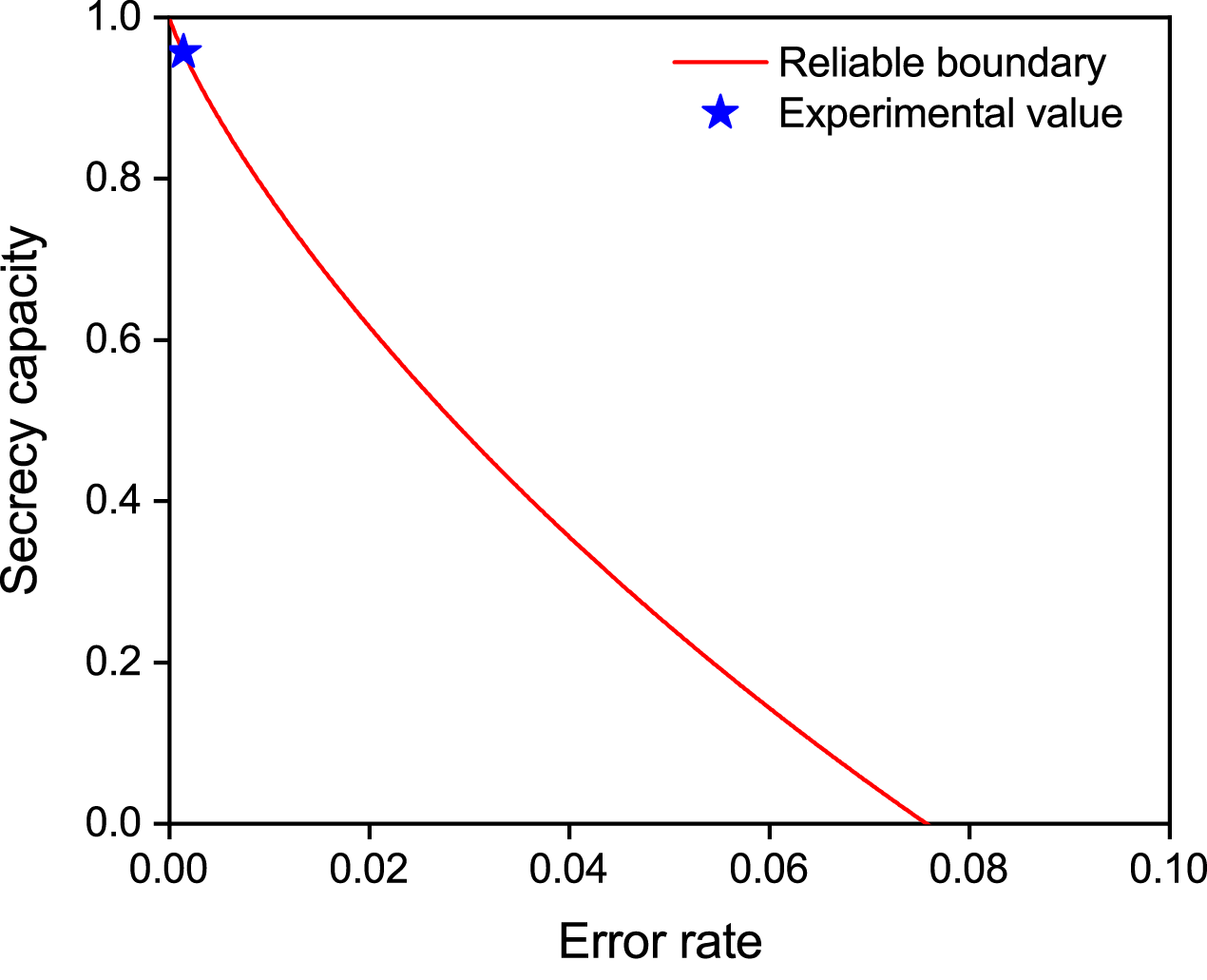


Fig. S2 | Secrecy capacity and error rate. The red dash line represents the boundary of the secure and insecure scenarios. The blue sign refers to the bit error rate obtained from the experiment. We assume that the response rate of the main channel and the wiretap channel is 1. The bit error rate threshold of the communication system is 0.076 under this wiretap channel model.

**Supplementary Reference**

1. Qi, R. *et al.* Implementation and security analysis of practical quantum secure direct communication. *Light*: *Science* *&* *Applications* **8**, 1-8 (2019).
2. Wyner, A. D. The wire‐tap channel. *The* *Bell* *System* *Technical* *Journal* **54**, 1355-1387 (1975).
3. Coles, P. J., Metodiev, E. M., & Lütkenhaus, N. Numerical approach for unstructured quantum key distribution. *Nature Communications* **7**, 1-9 (2016).
4. Holevo, A. S. Bounds for the quantity of information transmitted by a quantum communication channel. *Problemy Peredachi Informatsii*, **9**, 3-11(1973).
5. Ye, Z. D. *et al.* Generic security analysis framework for quantum secure direct communication. *Frontiers of Physics* **16**, 1-9 (2021).
